# Alexander Disease Modeling in Zebrafish: An In Vivo System Suitable to Perform Drug Screening

**DOI:** 10.3390/genes11121490

**Published:** 2020-12-11

**Authors:** Simona Candiani, Silvia Carestiato, Andreas F. Mack, Daniele Bani, Matteo Bozzo, Valentina Obino, Michela Ori, Francesca Rosamilia, Miriam De Sarlo, Mario Pestarino, Isabella Ceccherini, Tiziana Bachetti

**Affiliations:** 1 Department of Earth, Environment and Life Sciences (DISTAV), University of Genoa, 16132 Genoa, Italy; candiani@unige.it (S.C.); silvia.carestiato@edu.unige.it (S.C.); matteo.bozzo@edu.unige.it (M.B.); valentinaobino@gmail.com (V.O.); rosamilia.francesca@libero.it (F.R.); pesta@unige.it (M.P.); 2 Institut für Klinische Anatomie und Zellanalytik, Universitaet Tuebingen, 72076 Tuebingen, Germany; mack@anatu.uni-tuebingen.de; 3 Department of Clinical and Experimental Medicine, University of Florence, 50121 Florence, Italy; daniele.bani@unifi.it; 4 Department of Biology, University of Pisa, 56126 Pisa, Italy; michela.ori@unipi.it (M.O.); m.desarlo@studenti.unipi.it (M.D.S.); 5 Inter-University Center for the Promotion of the 3Rs Principles in Teaching & Research (Centro 3R), 56122 Pisa, Italy; 6 Laboratory of Genetics and Genomics of Rare Diseases, Unità Operativa Semplice Dipartimentale, Istituto Giannina Gaslini, 16147 Genoa, Italy; isa.c@unige.it

**Keywords:** Alexander disease, glial fibrillary acid protein, zebrafish, microinjection

## Abstract

Alexander disease (AxD) is a rare astrogliopathy caused by heterozygous mutations, either inherited or arising de novo, on the glial fibrillary acid protein (GFAP) gene (17q21). Mutations in the GFAP gene make the protein prone to forming aggregates which, together with heat-shock protein 27 (HSP27), αB-crystallin, ubiquitin, and proteasome, contribute to form Rosenthal fibers causing a toxic effect on the cell. Unfortunately, no pharmacological treatment is available yet, except for symptom reduction therapies, and patients undergo a progressive worsening of the disease. The aim of this study was the production of a zebrafish model for AxD, to have a system suitable for drug screening more complex than cell cultures. To this aim, embryos expressing the human *GFAP* gene carrying the most severe p.R239C under the control of the zebrafish *gfap* gene promoter underwent functional validation to assess several features already observed in in vitro and other in vivo models of AxD, such as the localization of mutant GFAP inclusions, the ultrastructural analysis of cells expressing mutant GFAP, the effects of treatments with ceftriaxone, and the heat shock response. Our results confirm that zebrafish is a suitable model both to study the molecular pathogenesis of *GFAP* mutations and to perform pharmacological screenings, likely useful for the search of therapies for AxD.

## 1. Introduction

Alexander disease (AxD) is a rare autosomal dominant astrogliopathy, characterized by progressively increasing severity and fatal outcome. AxD comprises two different clinical forms according to age at onset and decreasing progressive severity, namely, Type I AxD (early onset, <4 years of age) and Type II AxD (late onset, >4 years of age) [1]. Unfortunately, there is still no cure for this pathology; hence, therapies are only used to treat symptoms of the disease [2].

The vast majority of AxD patients are characterized by heterozygous mutations in the *GFAP* gene [3], formed of nine exons on chromosome 17q21 [4]. Its product is a 54 kDa type III intermediate filament protein, mainly expressed in the astrocytes of the central nervous system (CNS), involved in several astrocytic functions such as myelin maintenance, mitosis, and cell communication [5]. Although no correlation between genotype and phenotype has yet been found [6] the pathogenetic mechanism has been shown to rely on the inability of the mutant protein to correctly assemble into filaments, with a consequent formation of intracytoplasmic aggregates [2,7]. In addition to *GFAP* mutations, wildtype *GFAP* overexpression induces the formation of intracellular protein aggregates, thus suggesting that the gene expression level is a modifier element of the disease expressivity [6] and that regulation of *GFAP* expression may be a pharmacological target in AxD.

Indeed, the neuropathological hallmark of this astrogliopathy is the presence in patients’ astrocytes of Rosenthal fibers (RFs), intracytoplasmic inclusions containing wildtype and mutant glial fibrillary acid protein (GFAP), ubiquitin, proteasome components, and the two chaperone proteins αB-crystallin and heat-shock protein 27 (HSP27) [8].

Previous in vitro studies and in vivo experiments on mouse models showed that the increase in expression of HSP27 and αB-crystallin improves the p.R239C mutant protein folding [9]. According to these results, the use of the antibiotic ceftriaxone and the nutraceutical substance curcumin was shown to counteract the formation of aggregates in vitro by decreasing the level of mutant GFAP by inducing the HSP response and autophagy, in addition to decreasing *GFAP* transcription [10,11].

However, in vitro models suffer from limitations compared to animal organisms, with consequently arising difficulties in studying complex biological processes, such as those occurring in the nervous system that rely on interactions between different types of cells. On the other hand, mouse models are not easy to produce due to the long gestation time, and both managing and analysis of results suffer from complex procedures requiring specific expertise. Consequently, murine models are poorly suitable for high-throughput drug screenings. The use of a simpler vertebrate model, such as the zebrafish, can potentially overcome the above critical issues.

Zebrafish (*Danio rerio*) is a small tropical teleost fish often chosen as a model to study vertebrate biology because of its external development and rapid sexual maturity of only about three months. In addition, recombinant fusion fluorescent proteins can be used to study protein expression thanks to the optical transparency of larvae during embryogenesis [12]. Different studies showed that 71.4% of human protein-coding genes and 82% of human disease-associated genes are present in zebrafish [13]. Moreover, zebrafish is commonly adopted for studying nervous system development and setting up vertebrate models of monogenic neurodegenerative diseases [14]. During zebrafish gastrulation, the CNS originates from the neural plate and leads to the formation of the neural tube, via a process called secondary neurulation [15]. Zebrafish neurodevelopment is achieved through a set of distinct regulatory mechanisms evolutionarily conserved [16]. Regionalization and cell types of the zebrafish brain are not dissimilar from other vertebrates and contain different glial cell types such as the radial glia, oligodendrocytes, ependymal cells, and microglia. Moreover, in zebrafish, radial glia are not only present during development but are also the main glial cell type in the mature brain expressing *gfap*, while also maintaining stem-cell characteristics during the entire life cycle [17]. Radial glia are, thus, one of the most prominent glial cell types in the telencephalon; they supply proliferation and self-renewal, and they express similar embryonic genes in the adult [16]. This points to the possibility of a more specialized function of radial glial cells in fish, encompassing the functions of differentiated astrocytes [18].

In addition, several studies suggest a good homology between zebrafish and human *gfap* genes with a conserved intron–exon organization and transcriptional regulation. Indeed, the zebrafish *gfap* gene codes for a gfap protein which shares 67% identity and 77% homology with human GFAP [7]. Furthermore, it is well known that the gfap protein in zebrafish acts as an intermediate filament protein, supporting the suitability to study the human GFAP mutational effects in this animal model [4].

Taking as a starting point the previous observations reported by Lee et al. [7] involving zebrafish as a model of AxD, our work was aimed at improving and validating this model for further applications such as its suitability for high-throughput screening (HTS).

The validation of the model was performed by comparing the number of aggregates produced in GFAP wildtype (WT) and p.R239C zebrafish, by assessing the effects of ceftriaxone treatments and heat-shock protein stimulation in terms of aggregate formation.

## 2. Materials and Methods

Every procedure was performed in agreement with the Italian Ministry of Public Health legislation on the protection of animals used for scientific research, with authorization release n°69/2020-PR, pursuant to article 31 of the Legislative Decree of 26/2014. All the animal experiments involving zebrafish were approved in accordance with the local Ethic Committee on Animal Experimentation (protocol n°12804; approval date: 21 February 1997), pursuant to Legislative Decree n°10/1997-A of 31 January 1997.

### 2.1. Zebrafish Care and Breeding

Zebrafish adults were raised in a housing system with freshwater recirculation (water hardness between 400 and 800 μS/cm, temperature 28 °C, and pH 7.4). Each 3.5 L tank contained a number of animals ranging from 5–15. Zebrafish were fed three times a day with granular food (Zebrafeed, Sparos Ltd., Olhão, Portugal) and twice a week with *Artemia* nauplii.

Gametes were obtained by mating males and females in a 5 L tank, keeping them separated with a net for one night. The following day, they were put together in a new 5 L tank, separating all the fishes from the bottom with a net. After waiting 10–15 min for fertilization, eggs were collected in a 10 cm Petri dish with embryo medium (E3) [19].

### 2.2. Production of GFAP Plasmid for Transgenic Zebrafish

The pCS-TP, the pTol2-GFAP(p.R239C)-GFP, and pTol2-*gfap* promoter-GFP plasmids were kindly provided by Prof. Choi (Department of Biomedical Sciences and Neurology, Chonnam National University Medical School in Gwangju, Republic of Korea) [7].

The pTol2-GFAP WT plasmid was produced starting from the pcDNA3.1CT-GFP-TOPO-GFAP WT plasmid [20]. The GFAP-coding region was amplified by PCR forward and reverse primers containing the *EcoR*I (5′–AAATGAATTCGAGCCAGAGCAGG–3′) or *Sac*II (5′–CAACCCGCGGCATCACATCCTTGTGCTCCTG–3′) sites, respectively. The PCR product was subcloned in the pCR2.1 vector (TOPO TA cloning, Life Technologies, Monza, Italy) and then transferred in the pTol2 plasmid following *EcoR*I–*Sac*II digestion.

All Tol2 plasmids contained a GFP-coding region whose expression was regulated by the promoter of the zebrafish *gfap* gene, driving expression in the zebrafish glial cells. In the pTol2-*gfap* promoter-GFP plasmid there was not a GFAP-coding region, while, in the pTol2-GFAP WT-GFP and pTol2-GFAP (R239C)-GFP, there was the wildtype or the mutant GFAP-coding region, respectively.

The Tol2 transposon is a system used to express exogen proteins in zebrafish since it is active in all vertebrate cells, including human [21]. Tol2 system functionality requires the coinjection of the capped messenger RNA (mRNA) encoding the Tol2 transposase protein responsible for the insertion of the exogenous sequence in the zebrafish genome. The pCS-TP plasmid, containing the Tol2 transposase coding sequence, was linearized and used as a template for the in vitro synthesis of pCS-TP capped mRNA using the mMESSAGE mMACHINE^®^ Kit (Thermo Fisher Scientific, Monza, Italy), according to the manufacturer’s instructions. Tol2 capped mRNA was precipitated with 3 M sodium acetate pH 3.5, resuspended in RNAse-free water, and stored at −80 °C [22].

### 2.3. Microinjection, Embryo Cultures, and Phenotype Evaluation

Microinjection is a useful technique to achieve transgenic animals as models of study. Embryos were microinjected at the one-cell stage (0–0.5 h post fertilization, hpf) with 0.5 nL of a mix solution containing 280 ng/μL of a GFAP expression construct, 280 ng/μL Tol2 transposase capped mRNA, and 10% phenol red using FemtoTip II capillaries (Eppendorf, Milan, Italy), an MP-1 micromanipulator (Narishige Group, London, UK), and a FemtoJet^®^ microinjector (Eppendorf, Milan, Italy).

After microinjection, embryos were raised at 28 °C in E3 overnight. The following day (at 24 hpf), 0.003% of PTU (1-phenyl 2-thiourea) was added to the cultures to inhibit melanogenesis. The effects of transient *GFAP* overexpression were evaluated at 24 hpf with an Olympus IX70 epifluorescence microscope equipped with a ColorView II camera and interfaced with AnalySIS software (Soft Imaging System GmbH, Münster, Germany). The GFAP aggregate counting was achieved with ImageJ 1.52q software [23].

### 2.4. Immunofluorescence Assays

At 2 days post fertilization (dpf), mutant embryos were anesthetized with tricaine (MS222) (Sigma-Aldrich, Milan, Italy) and fixed with 4% paraformaldehyde in phosphate buffered saline (PBS) overnight at 4 °C. Fixed embryos were washed in PBS, embedded in optimal cutting temperature compound (OCT), and frozen in liquid nitrogen. Sections of 10 μm thickness were obtained with a Leica CM1900UV cryostat (Leica, Buccinasco, Italy). Sections were thawed at room temperature, washed in PBS, and incubated first with blocking solution (10% goat serum + 0.1% Triton X-100 (Sigma-Aldrich, Milan, Italy) for 2 h at room temperature and subsequently with a primary antibody diluted 1:100 in blocking solution at 4 °C overnight. Either rabbit anti-human GFAP (ab7260) (Abcam, Milan, Italy) or mouse anti-human GFAP (#3670) (Cell Signaling, Milan, Italy) was used. The following day, sections were washed with several changes in PBS and incubated with secondary antibody diluted 1:1000 in blocking solution for 20–25 min. Alexa Fluor^®^ 555 anti-rabbit (A21428) (ThermoFisher, Monza, Italy) and anti-mouse (A11031) (ThermoFisher, Monza, Italy) were used as secondary antibodies, while 10 μg/mL Hoechst 33342 (14530) (Sigma-Aldrich, Milan, Italy) was used as nuclear counterstain. Sections were imaged with either a Leica TCS SP5 AOBS confocal microscope (Leica Microsystems, Mannheim, Germany) or an Olympus IX70 epifluorescence microscope (Olympus, Segrate, Italy). ImageJ 1.52q (Bethesda, MD, USA) was used to elaborate confocal images. Adobe^®^ Photoshop (San Jose, CA, USA) was used to perform minor adjustments and to assemble individual figures into tables.

### 2.5. Statistical Analysis

Data quantification was performed with ImageJ 1.52q software and processed with Microsoft Office^®^ Excel (Redmond, WA, USA) on histograms and Gaussian distributions. A *p*-value was established with one-way ANOVA, and all reported results were considered as statistically significant for *p*-values < 0.05.

### 2.6. Ceftriaxone Treatments

Microinjected embryos were treated in a 24-well plate with 1.0 mM of the antibiotic ceftriaxone (CEF) diluted in E3 containing 0.003% PTU. After 24 and 48 h of treatment, embryos were analyzed with an Olympus IX70 fluorescence microscope (Olympus, Segrate, Italy) and photographed with the interfaced camera. Images were processed with ImageJ 1.52q (Bethesda, MD, USA) and Adobe^®^ Photoshop (San Jose, CA, USA).

### 2.7. Heat-Shock Protein Stimulation

At 24 hpf, GFAP R239C-GFP-injected embryos were subjected to thermal shock by incubation in a thermostatic bath at 37 °C for 1 h [24]. Next, thermally shocked embryos were incubated at 28 °C and analyzed with an Olympus IX70 epifluorescence microscope (Olympus, Segrate, Italy) at 3 and 24 h from the thermal shock. Shocked embryos were compared with unshocked mutant embryos of the same age. Images were processed with Adobe^®^ Photoshop (San Jose, CA, USA).

### 2.8. Transmission Electron Microscopy

Zebrafish larvae were fixed in 4% glutaraldehyde in 0.1 M cacodylate buffer (pH 7.4) for 24 h and post-fixed in 1% osmium tetroxide in 0.1 M phosphate buffer (pH 7.4) for 1 h. Subsequently, fixed larvae were dehydrated in graded acetone and embedded in Epon 812 epoxy resin (Fluka, Zurich, Switzerland). Ultrathin sections were stained with 1% samarium triacetate–10% gadolinium triacetate (UAR-EMS stain #22405, Electron Microscopy Sciences, Roma, Italy) counterstained with lead citrate and examined under a JEM 1010 electron microscope (Jeol, Freising, Germany) at 80 kV.

Alternatively, zebrafish larvae were fixed in 2.5% glutaraldehyde in 0.1 M cacodylate buffer (pH 7.4), for 2 h at room temperature and post-fixed in 1% osmium tetroxide in PBS, followed by dehydration in graded ethanol series. For contrast enhancement, the 70% ethanol was saturated with uranyl acetate. Dehydration was completed by acetone, followed by propylene oxide. Specimens were infiltrated with rising concentrations of epoxy embedding medium (Sigma-Aldrich, Milan, Italy). Ultrathin sections were cut on a Leica Ultramicrotome (Leica Microsystems, Mannheim, Germany) and mounted on pioloform-coated copper grids. Ultrathin sections were examined and documented using a Zeiss EM10 or a LEO 912AB transmission electron microscope (both from Carl-Zeiss-Stiftung, Oberkochen, Germany). Images were captured with a digital camera using ImageSP software (Minsk, Belarus, Russian) and assembled in tables with Adobe^®^ Photoshop (San Jose, CA, USA).

## 3. Results

### 3.1. Localization and Quantification of GFAP Aggregates

**Figure 1:**
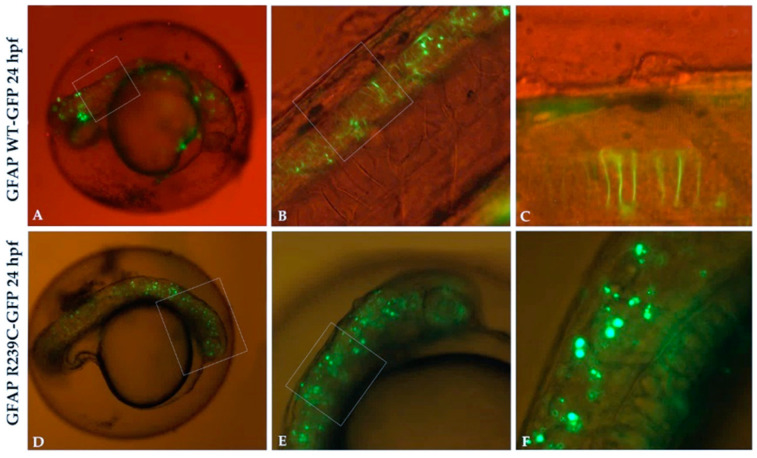
GFP expression in transgenic glial fibrillary acid protein (GFAP) wildtype (WT) and GFAP R239C embryos. (**A**,**B**) The expression of GFAP WT-GFP protein is visible along the neural tube, with very few GFAP aggregates observed. (**C**) High magnification of embryo in (**B**) shows GFAP WT protein as filamentous structures. (**D**–**F**) Several aggregates of p.R239C in the brain and along the neural tube are found. Magnification: 4× (**A**,**D**); 10× (**B**,**E**); 20× (**C**,**F**). High magnification are shown by rectangular shape.

One of the most severe *GFAP* mutations recognized as causative of AxD is the missense p.R239C, which we used to create a zebrafish model of the disease. To this purpose, we took advantage of the Tol2 approach, reported to have the highest rate of genomic integration in the germ lineage and widely used as a genetic tool [25]. We compared embryos microinjected with plasmid constructs expressing GFAP R239C-GFP and GFAP WT-GFP. At 24 h, GFAP WT-GFP embryos showed a widespread expression of GFAP proteins along the entire neural tube with most of these in the form of filamentous structures (Figure 1A–C) as the correct folding of the protein, even if some GFAP aggregates appeared. This was due to an overexpression of the protein following microinjection and, as a consequence, the protein was prone to aggregation. This result was also reported in several mouse models [26] and cell lines transfected with GFAP constructs [20,27]. In embryos microinjected with the construct GFAP R239C-GFP (Figure 1D–F), the protein p.R239C was visible in the form of aggregates along the entire neural tube. Unlike GFAP WT-GFP embryos, the p.R239C mutation induced a significantly higher number of aggregates, despite some filamentous structures being present, albeit to a minor extent.

**Figure 2:**
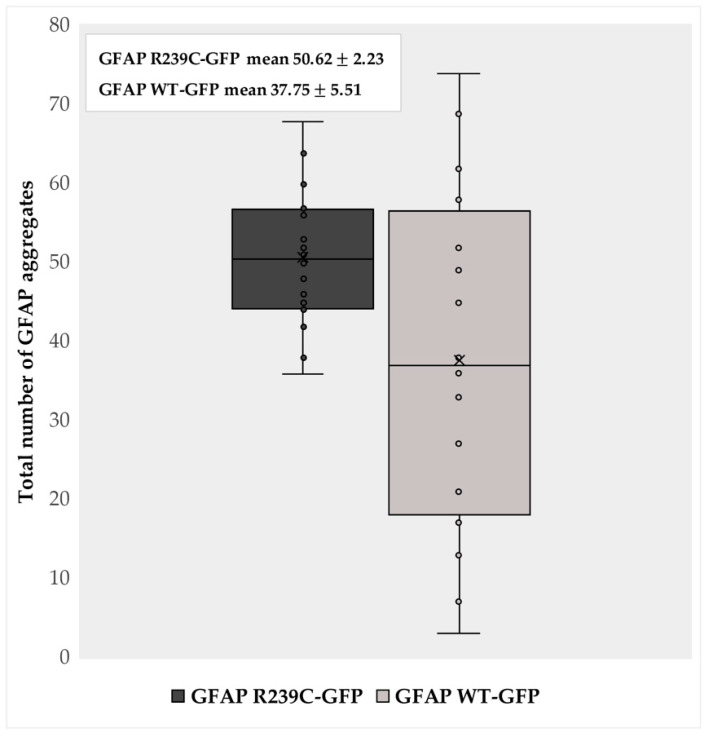
Aggregate quantification. The box plot represents the number of aggregates detected in 16 GFAP R239C-GFP embryos and in 16 GFAP WT-GFP embryos at 24 h post fertilization (hpf). The “x” indicates the average values of 50.62 and 37.75 aggregates in p.R239C and wildtype embryos, with a standard error of the mean (SEM) of ±2.23 and ±5.51, respectively. The count of the aggregates was made using ImageJ software 1.52q.

After evaluating the localization, aggregates were quantified using the ImageJ 1.52q software. A comparison at 24 hpf between 16 GFAP WT-GFP-microinjected embryos and 16 embryos expressing mutant p.R239C was performed, and the results are summarized in the box plot reported in Figure 2 (aggregate details for each embryo are reported in Figure S1 (Supplementary Materials). The presented results highlighted the high number of aggregates in each GFAP R239C-GFP embryo, ranging from 36 to 68, with an average value of 50.62 and a standard deviation (SD) of ±8.94. Instead, the quantification of GFAP WT-GFP embryos underlined the presence of a highly variable number of aggregates, ranging from 3 to 74, with a median value of 37.75 and an SD of ±22.04. Despite the maximal number quantified, the data of GFAP WT-GFP embryos did not show significant differences compared to the data in GFAP R239C-GFP, derived from the mutation. By comparing the two obtained means, a *p*-value of 0.038 was determined.

**Figure 3:**
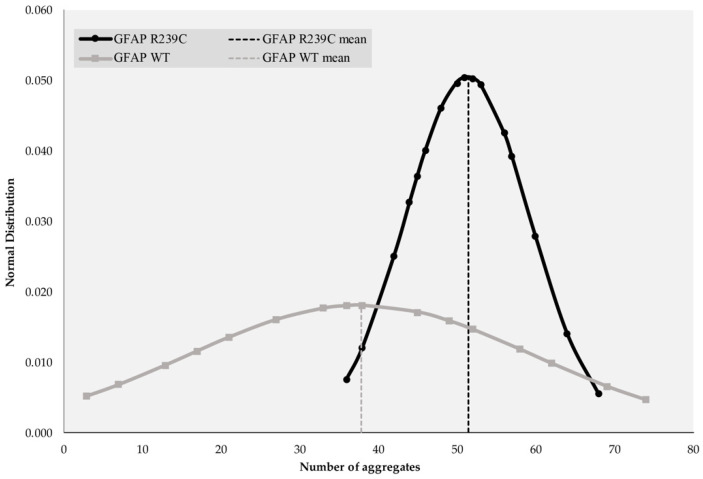
Gaussian distribution of GFAP R239C-GFP and GFAP WT-GFP aggregates. The number of aggregates and normal distribution are reported on the abscissa and ordinate axes, respectively. A *p*-value of 0.038 was found and standard deviations of ±8.65 and ±22.04 were obtained for GFAP R239C-GFP and GFAP WT-GFP, respectively. Regarding quantification, a mean of 50.62 aggregates was found for GFAP R239C-GFP embryos, while a mean value of 37.75 aggregates was established for GFAP WT-GFP ones. For GFAP R239C-GFP and GFAP WT-GFP embryos, medians of 50.5 and 37 were found.

On the basis of the derived mean and standard deviation values of the aggregates detected, Gaussian probability distribution curves were constructed for GFAP WT-GFP and GFAP R239C-GFP embryos. Looking at Figure 3, the amplitude of the curves highlighted the different deviations from the mean value, which were higher in GFAP WT-GFP versus GFAP R239C-GFP embryos. We can deduce that the mutation showed a reproducible trend.

### 3.2. Localization of Mutant GFAP Aggregates

**Figure 4:**
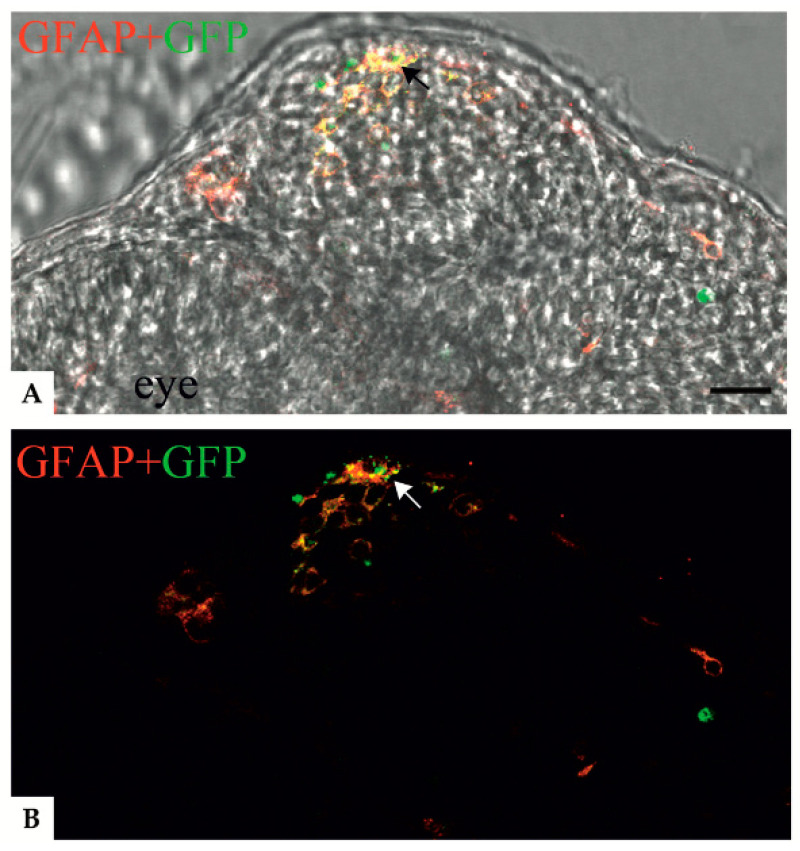
Confocal images showing colocalization between anti-GFAP signal and the GFAP R239C-GFP aggregates in zebrafish transverse sections of 24 hpf embryos. (**A**,**B**) Confocal images showing p.R239C aggregates (green) labeled by anti-GFAP antibody (red). A clear colocalization (yellow) (black and white arrows respectively in A and B) is visible in most of the cells containing green aggregates. (**A**) Confocal image imposed on a brightfield image of the same region. Scale bar: (**A**,**B**) 30 μm.

Immunofluorescence analyses, performed on embryos expressing GFAP p.R239C, allowed us to determine the intracellular localization of anti-GFAP and GFAP R239C-GFP signals. In particular, confocal microscope analysis of cells in the region of the CNS in proximity of the eye showed that p.R239C aggregates (Figure 4A) were characterized by reduced dimensions compared to the cell nucleus, with the latter defined in the image by the empty compartment surrounded by red fluorescence in Figure 4B, thus identifying green dots as intracellular inclusions.

**Figure 5:**
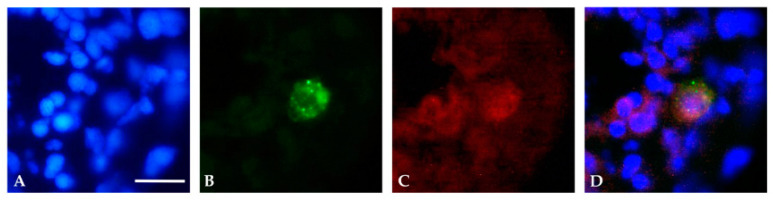
Immunofluorescence analysis of transverse sections of a 24 hpf embryo. (**A**) Nuclei were detected with Hoechst (blue). (**B**) p.R239C expression (GFP-green), (**C**) identified by anti-human GFAP antibody (red). (**D**) Representation of the overlap of the three images. Scale bar (**A**–**D**): 20 μm.

Moreover, such a finding was further confirmed by immunofluorescence analysis using an anti-hGFAP antibody. Specifically, colocalization of green p.R239C-GFP GFAP and red anti-hGFAP signals confirmed that green fluorescence was represented by GFAP expression and that aggregates were inside the cytoplasm of cells (Figure 5).

### 3.3. Effects of Ceftriaxone Treatments

To further validate the zebrafish model of AxD, we investigated whether the antibiotic ceftriaxone (CEF), previously shown to decrease mutant GFAP aggregation in vitro [10] and to improve the clinical severity in an AxD patient [28], could also exert beneficial effects in our in vivo model.

First, titration tests were performed by treating wildtype embryos for 24 and 48 h to determine the safe range of CEF concentration to use in vivo. The effects of CEF treatments were evaluated analyzing embryo morphology. As shown in Figure S2 (Supplementary Materials), no toxic effects on embryos were reported at 100 μM, 1.0 mM, or 1.5 mM, or in its ethanol diluent at 15 μM, 25 μM, or 40 μM.

Then, we selected a set of GFAP p.R239C microinjected embryos carrying a number of aggregates greater than 50. In each experiment, about half of them were used as untreated controls and half were treated with ceftriaxone 1.0 mM for 48 h.

**Figure 6:**
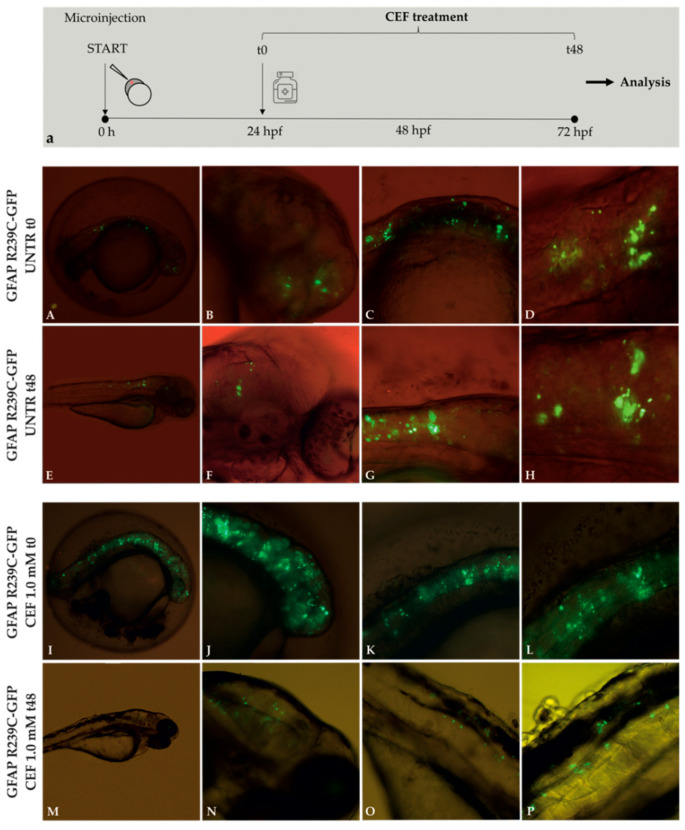
Experimental plan showing the timeline of ceftriaxone (CEF) treatments and the effects of 48 h CEF 1.0 mM treatment on embryos expressing GFAP R239C-GFP. (**a**). The antibiotic ceftriaxone was added 24 h after eggs microinjection. This moment was defined as “time zero” (t0). Fluorescence microscope analysis. Aggregates in untreated (UNTR) embryos are shown in figure (**A**–**D**) at 24 hpf (t0) and from (**E**–**H**) at 72 hpf (t48), corresponding to the start and end points of CEF treatment. Figure (**I**–**L**) show aggregates at 24 hpf (t0), while figure (**M**–**P**) show aggregates at 72 hpf (t48), after 48 h of treatment with CEF 1.0 mM. Magnification: 4× (**A**,**E**,**I**,**M**); 10× (**B**,**C**,**F**,**G**,**J**, **K**,**N**,**O**); 20× (**D**,**H**,**L**,**P**).

Following 48 h of treatment with CEF 1.0 mM, a decrease or complete disappearance of aggregates occurred in the vast majority of treated p.R239C embryos (Figure 6), which we defined as “rescued”. This result was not a time-dependent effect since 80% untreated p.R239C embryos did not show any decrease in the number of aggregates during the time of treatment of the other group, thus suggesting that inclusions were stable along time (Table S1, Supplementary Materials).

**Figure 7:**
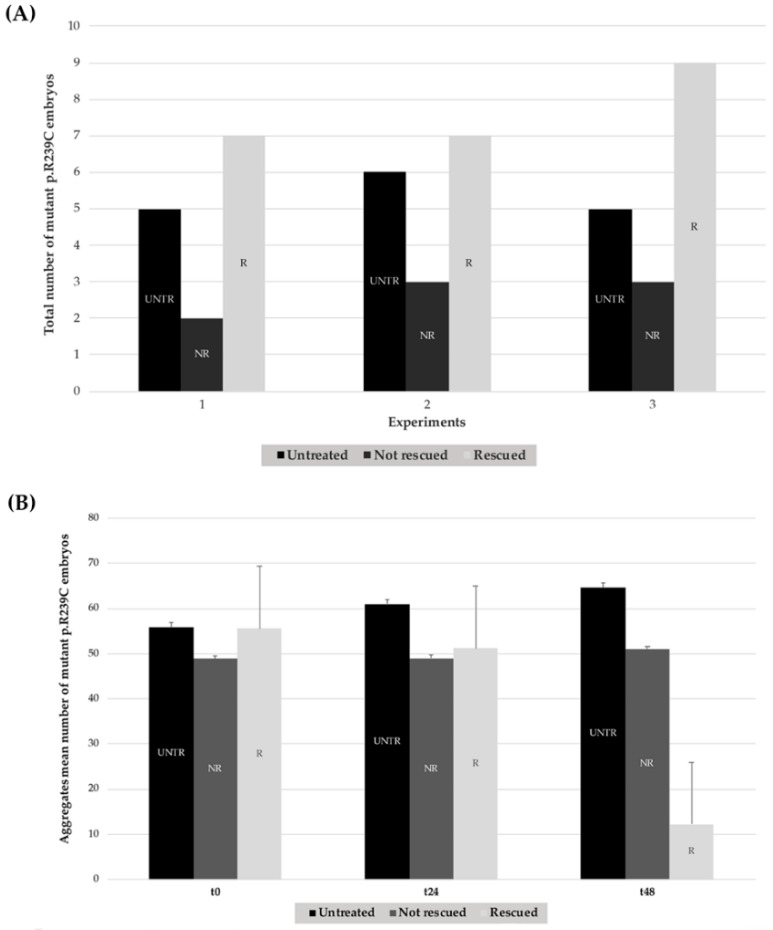
Effects of treatments with CEF 1.0 mM on mutant GFAP R239C-GFP-injected embryos in three explicative experiments. (**A**) In each histogram, the black column represents the number of untreated embryos (UNTR) compared to the not rescued in the dark-gray column (NR), not showing effects in terms of aggregate reduction, and the rescued (R) ones, in the light-gray column, which responded to pharmacological treatment with consequent aggregate reduction. For “R” embryos, a paired *t*-test two-tailed *p*-value of 0.0075 was determined. (**B**) The mean number of aggregates is reported for untreated, not rescued, and rescued embryos at t0, t24, and after 48 h (t48). For “R” embryos, a paired *t*-test two-tailed *p*-value of 0.0019 was achieved. (UNTR: untreated R: rescued, NR: not rescued).

Treatments with CEF 1.0 mM on mutant embryos were performed in triplicate and showed that the antibiotic could rescue the phenotype in 74.2% of embryos. On the contrary, no decrease in the number of aggregates was observed in untreated embryos (Figure 7, Table 1, Table 2 and Table 3).

Moreover, to determine that the effect of CEF on the reduction of GFP inclusions was not specific but rather due to post-transcriptional or post-translational regulation of *GFAP* expression, five GFAP WT-GFP embryos were treated with CEF under the same experimental conditions and compared with untreated WT embryos. As shown in Table S2 (Supplementary Materials), unlike that observed for mutant embryos, a reduction in aggregates was observed in 35–40% WT embryos after 48 h, but independently of treatment. Such results could be ascribed to cellular responses able to counteract such inclusions in a less damaging context than cells expressing the severe GFAP mutation. As GFAP aggregation seemed to be correlated with gene expression levels [29] and CEF was demonstrated to decrease *GFAP* expression by downregulating *GFAP* transcription in cellular models [10], we investigated whether the beneficial effect on mutant GFAP aggregation could be partially ascribed to a reduction in protein level due to decreased zebrafish *gfap* promoter activity.

**Figure 8:**
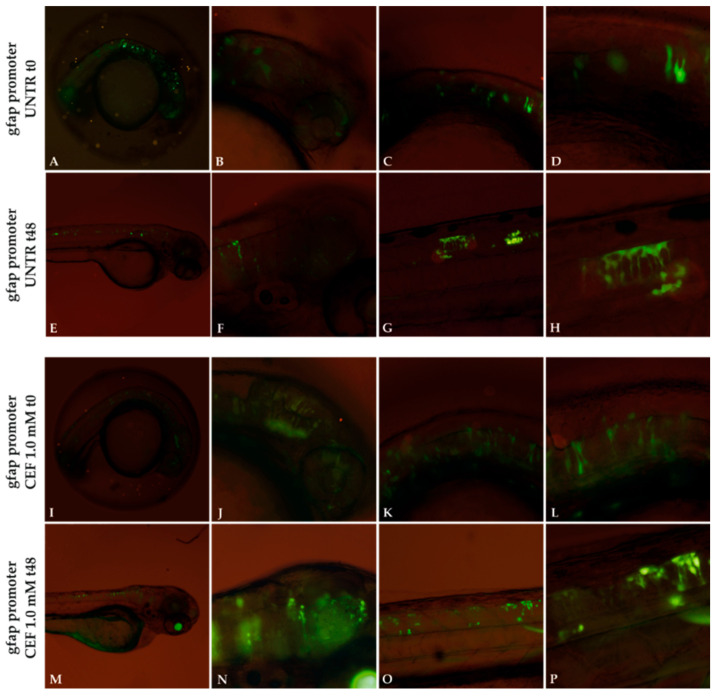
Effect of 1.0 mM CEF treatment on *gfap* promoter. Untreated (UNTR) embryos expressing *gfap* promoter as filamentous structures (**A**–**H**) at 24 and 72 hpf, respectively. CEF effects on embryos at t0 (24 hpf) (**I**–**L**) and after 48h of treatments (**M**–**P**). Magnification: 4× (**A**,**E**,**I**,**M**); 10× (**B**,**C**,**F**,**G**,**J**,**K**,**N**,**O**); 20× (**D**,**H**,**L**,**P**).

First, the analysis of embryos injected with the pTol2-*gfap* promoter-GFP construct, lacking any GFAP-coding region, showed an expression pattern of GFP corresponding to that reported by Bernardos et al. [30], thus confirming that GFP was correctly expressed at this stage (Figure 8). The analysis of the intensity of the fluorescence before (t0, Figure 8I–L) and after 48 h of treatment with CEF 1.0 mM (Figure 8M–P) did not highlight any effect of the drug on *gfap* promoter compared to untreated embryos (Figure 8A–H) at the same time of exposure, thus excluding that the decrease in mutant GFAP aggregates could be ascribed to a decrease in gene expression. Analysis at 24 h is reported in Figure S3 (Supplementary Materials).

### 3.4. Stimulation Effects of the Small Heat-Shock Proteins

Heat shock was reported to induce the expression of small heat-shock proteins (sHSPs) in zebrafish, including HSPB1, which encodes for the homolog of human HSP27 [31]. Following such observations, we investigated whether sHSP induction, produced by heat-shock stimulation, could also have beneficial effects on mutant GFAP aggregates in zebrafish embryos.

After selecting a set of GFAP p.R239C microinjected embryos carrying a number of aggregates greater than 50, about half of them were used as untreated controls and half were shocked for 1 h at 37 °C.

**Figure 9:**
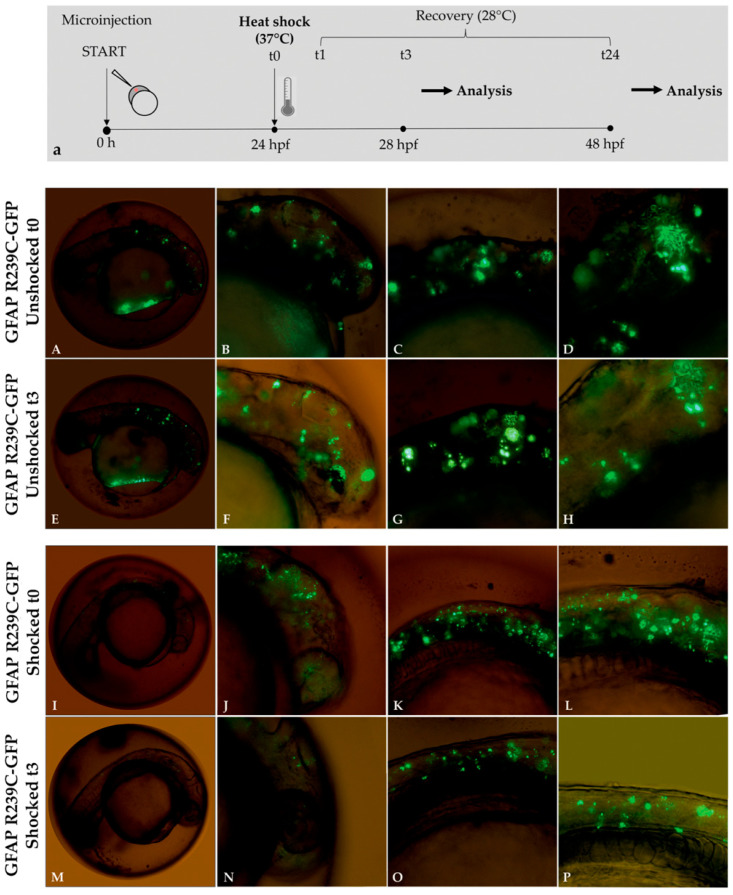
Experimental plan showing the timeline of heat-shock treatments and effects of thermal shock on p.R239C aggregates. (**a**) At 24 hpf, embryos were incubated for 1 h at 37 °C and then recovered at 28 °C. Fluorescence microscope analysis was performed after 3 (t3) and 24 h (t24) from the end of the 1 h heat shock (t1). In Figure (**A**–**H**), mutant embryos not shocked (unshocked) are presented at 24 hpf, showing diffuse aggregate formation before and after 3 h. Figure (**I**–**L**) show GFAP p.R239C aggregates in embryos at 24 hpf, while, in figure (**M**–**P**), the thermal shock effect is shown after 3 h on GFAP p.R239C aggregates. Magnification: 4× (**A**,**E**,**J**,**M**); 10× (**B**,**C**,**F**,**G**,**J**,**K**,**N**,**O**); 20× (**D**,**H**,**L**,**P**).

Analyses performed on p.R239C embryos after 3 h from thermal shock showed that the number of GFAP aggregates was reduced in thermally shocked mutant embryos compared to nonshocked (unshocked) mutant embryos of the same age (Figure 9).

Analysis performed at 24 h post treatment showed that the heat-shock response effect was transient, as aggregates started increasing again (Figure S4, Supplementary Materials).

**Figure 10:**
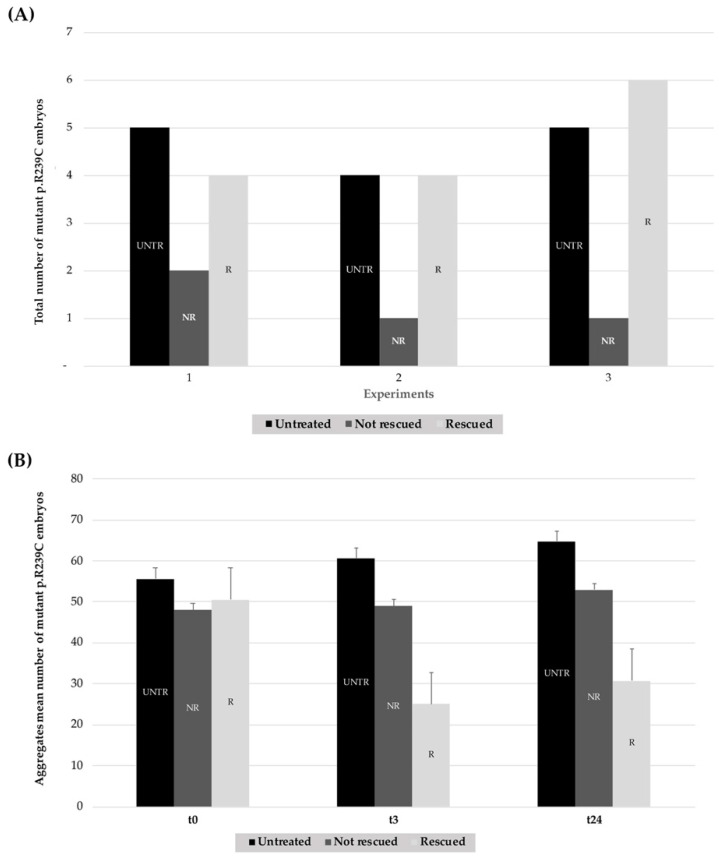
Summary histograms showing the effect of heat-shock treatments on GFAP R239C-GFP-injected embryos from three replicates. (**A**) For each experiment (n° 1–3), the lighter column of each series (R) represents the number of embryos showing a reduction in p.R239C aggregates, compared to the untreated embryos (UNTR) and the not rescued (NR) ones, the latter in which the thermal shock did not result in aggregate reduction. For “R” embryos, a paired *t*-test two-tailed *p*-value of 0.0022 was determined. (**B**) The respective count of aggregates is reported in the darker column for untreated embryos (UNTR), compared to the not rescued (NR) and the rescued (R) ones at t0 and after 3 (t3) and 24 h (t24). For “R” embryos, a paired *t*-test two-tailed *p*-value of 0.0048 was found. (UNTR: untreated, R: rescued, NR: not rescued).

The quantification of the effects of thermal shock on GFAP R239C-GFP embryos is shown in Figure 10. The respective percentages of rescued embryos for each experiment, thus resulting in a total of 14/18 rescued embryos showing a reduction in p.R239C aggregates (Table 4), in addition to the respective count of aggregates (Table 5 and Table 6). On the contrary, embryos not shocked did not show any rescue, thus confirming that the aggregate decrease was not time-dependent but heat-shock-induced.

This result confirmed that the induction of sHSPs was also positively involved in the reduction of GFAP p.R239C aggregates in our zebrafish model of AxD, providing a mechanism of protection against protein aggregation in zebrafish cells and strengthening the suitability of the model.

### 3.5. Ultrastructural Analysis of the Zebrafish Model of AxD

To provide further validation of zebrafish as a suitable model for studying AxD, detailed investigations on aggregate p.R239C localization, already observed in fluorescence, were performed using transmission electron microscopy.

**Figure 11:**
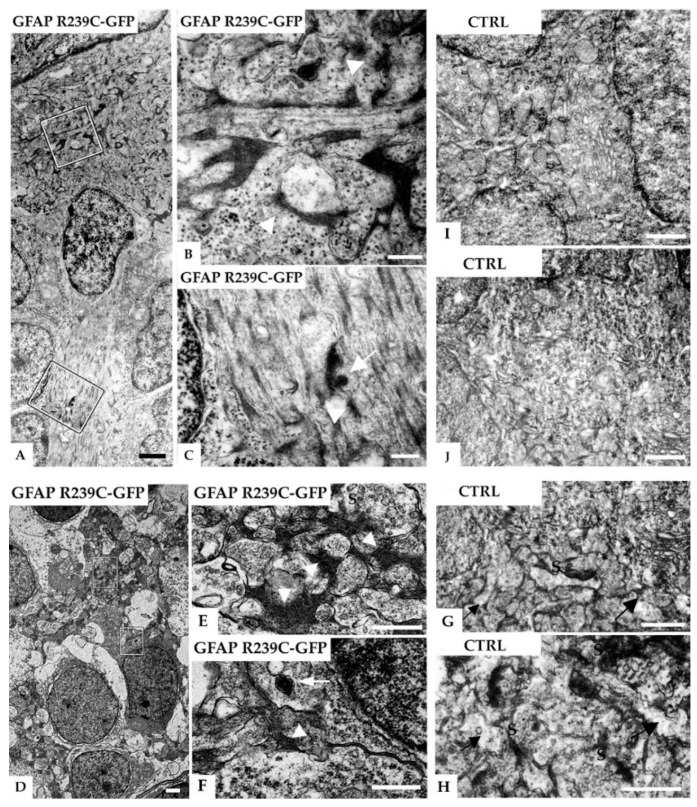
Electron micrographs of radial glial cells in the subventricular and neuropil region in the telencephalon of a 5 dpf zebrafish injected with GFAP-R239C-GFP plasmid. (**A**) A glial cell contacts the ventricular brain surface covered by the tela choroidea (top) and shows many electron-dense profiles. In (**B**,**C**), higher magnifications of the marked areas in (**A**) reveal that the dark profiles are associated with filaments (arrowheads) and sometimes surrounded by a membrane (arrows), the latter consistent with lysosomal packaging. In another example of the neuropil region shown in (**D**), at higher magnifications of the marked areas depicted in (**E**,**F**), glial processes containing electron-dense fibrillar material can be seen (arrowheads). The arrow points at a membrane-bound vesicle containing clustered electron-dense material, featuring a lysosome. In (**G**–**J**), electron microscopic analyses on control wildtype embryos of similar regions are shown, i.e., subventricular glial processes in (**G**,**H**) and neuropil region in the telencephalon in (**I**,**J**). No such electron-dense material in glial processes, identified by their irregular shape in the synaptic regions (arrows), was detected in the control tissue. Note that the dark bands are postsynaptic densities (S: presynaptic terminals). Scale bars: (**A**,**D**) 1 µm; (**B**,**C**) 250 nm; (**E**–**J**) 500 nm.

Images of radial glial cells from a 5 dpf GFAP R239C-GFP embryo showed electron-dense material in the cytoplasmatic compartment (Figure 11A). In addition to electron-dense areas and fibrillary structures, membrane-bound vesicles containing electron-dense material can be observed, likely representing lysosomes engulfing GFAP dense material (Figure 11B,C). The same observation was detected in the neuropil region, where some darker areas of amorphous material, contained in membrane-bound cell structures, were observed (Figure 11B–F).

Our results from electron microscopy suggested how mutant GFAP p.R239C protein was prone to aggregation, while it was driven to the lysosomal pathway and, thus, likely to partial protein elimination as a cellular response to toxic inclusions. Analysis of embryos expressing GFAP-WT-GFP protein is reported in Figure S5 (Supplementary Materials).

**Figure 12:**
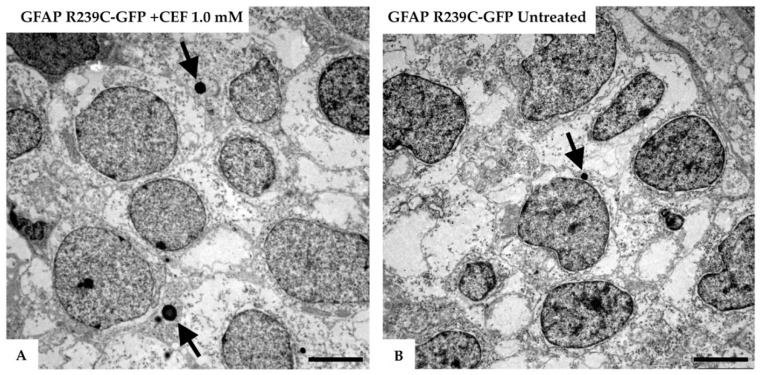
Electron micrographs of the telencephalon of 3 dpf zebrafish embryos expressing GFAP R239C-GFP.(**A**) The formation of lysosomes (arrows) was confirmed in the electron micrographs of the telencephalic region from a 3 dpf old zebrafish, injected with GFAP R239C-GFP plasmid and treated with 1.0 mM ceftriaxone for 48 h. (**B**) In contrast, in mutant untreated embryos, fewer and smaller lysosomes were seen at this embryonic stage. Images were captured with Zeiss EM10 or a LEO 912AB transmission electron microscope. Scale bar: 2.5 µm.

In addition, the analysis of embryos treated with CEF showed an increase in the formation of lysosomes (Figure 12), in accordance with the increased autophagy observed in vitro [10].

## 4. Discussion

Alexander disease (AxD) is a rare leukodystrophy that is only symptomatologically treatable to date [2]. Although cellular and animal models have been set up to study the underlying molecular mechanisms [32], so far, no animal model suitable to perform a large-scale drug screening has been established.

In the present study, we reported an improvement in the generation and validation of a zebrafish model for AxD expressing one of the most severe mutations in humans, the missense p.R239C, previously observed and described by Lee et al. [7]. By using the microinjection technique, we observed morphological differences between p.R239C mutant embryos and those expressing WT GFAP protein. Our results highlighted the formation of GFAP aggregates in most p.R239C mutant embryos along the neural tube, while filamentous structures were predominant in WT GFAP mutants. Ultrastructural analyses confirmed the morphological pattern observed with fluorescence investigations. In particular, in addition to the observation of anomalous dense material not detected in WT embryos, this suggested that the lysosome pathways are likely responsible for the partial elimination of mutant GFAP aggregates.

Moreover, results shown in this study confirmed reproducible patterns and constant differences between embryos expressing GFAP R239C and wildtype proteins.

In addition, the presence of GFAP aggregates was verified with the colocalization of anti-GFAP and GFAP R239C-GFP signals in astroglial cells, confirming that mutant GFAP expression was correctly driven by the *gfap* promoter.

To further validate the zebrafish model for AxD, we evaluated in vivo the effects of ceftriaxone (CEF) antibiotic, previously shown to be efficient in reducing GFAP aggregates in in vitro models [10,11].

Mutant embryos treated for 48 h with CEF 1.0 mM confirmed the beneficial effect of the antibiotic on the GFAP R239C aggregates in vivo. Such an effect was demonstrated not to be dependent on the downregulation of GFAP expression, suggesting that it was likely due to an improvement in folding of the mutant protein. Unlike that observed in vitro on the promoter of the human GFAP gene, CEF was not able to regulate GFAP expression by acting on the zebrafish *gfap* promoter. This result was not unexpected as, outside of the coding region, conservation between human and fish regulatory regions is low and likely differs in the elements regulated by CEF.

Our ultrastructural analysis confirmed the beneficial effects of CEF 1.0 mM on embryos expressing GFAP p.R239C, as many lysosomes containing dense material were detected, likely involved in the clearance of mutant GFAP. Lysosomes were also identified in untreated embryos expressing p.R239C, a presence that may be linked to the activation of cellular autophagy, a known cellular response to proteasome overloading caused by mutant GFAP [33]. These results not only provided further validation of the zebrafish model by reproducing the effects of the drugs observed in cell cultures and in a patient [10,28], but also confirmed that ceftriaxone is worthy of further investigation as therapy.

One of the cellular mechanisms acting to ensure the correct folding of proteins and their degradation involves the small heat-shock proteins (sHSPs). The role of the heat-shock response in AxD was strongly confirmed by several observations. Indeed, the overexpression of HSP27 and αB-crystallin by transient transfection in cells expressing mutant GFAP or following treatments with curcumin [11] or ceftriaxone [10] showed a reduction in aggregates, due to induction of mutant protein elimination by autophagy. Moreover, αB-crystallin overexpression also showed a beneficial effect in murine models overexpressing mutant GFAP [11]. Heat-shock induction in zebrafish embryos carrying p.R239C mutant GFAP resulted in a marked reduction of aggregates. This suggested that stimulation of the heat-shock response likely mediated by the sHSPs could also exert beneficial effects in zebrafish, thus supporting zebrafish expressing mutant p.R239C GFAP as a reliable model for AxD.

However, for complete validation of AxD in zebrafish, further work is required addressing various aspects of the pathology, for instance, demyelination. This aspect of the disease is particularly challenging because, so far, no mouse models, despite developing Rosenthal fibers and astrocyte reactivity, can recapitulate white-matter abnormalities or reductions in survival.

On the other hand, we are aware that our transient model of zebrafish presents some limitations in terms of studying the neurodegenerative development of AxD in the adult. Rather than a mosaic model, the generation of a transgenic stable line could be more suitable for further investigating the pathogenesis of late-onset AxD.

Nevertheless, as most AxD cases present with infantile or juvenile onset, due to transparency during embryogenesis and early life, zebrafish provide a very suitable model system to observe the development of the disease in early onset, as well as to perform morphological and functional studies, in addition to drug screening to search for specific therapy.

## 5. Conclusions

In this study, we demonstrated how zebrafish can be a valid model to reproduce the GFAP p.R239C mutation of Alexander disease in vivo. We confirmed the glial localization of aggregates using immunocytochemistry and electron microscopy. Furthermore, our results showed the positive effects on mutant p.R239C embryos of ceftriaxone treatments and sHSP stimulation in terms of GFAP aggregate reduction. Our results globally support the use of zebrafish as a powerful model for the study of the molecular pathogenesis of Alexander disease and for high-throughput drug screenings for future therapies.

## Figures and Tables

**Table 1:** The table shows data obtained in three treatments with ceftriaxone 1.0 mM, comparing the total number of treated embryos (“treated”) with the rescued ones (“rescued”) versus the untreated (“untreated”). The rescued are expressed as a percentage (“% rescue”). Percentages of mean value and standard deviation are shown at the bottom of the table.

Number of Mutant p.R239C Embryos Treated with CEF 1.0 mM				
Experiment	Untreated	Treated	Rescued	% Rescued
n°1	5	9	7	77.70%
n°2	6	10	7	70.00%
n°3	5	12	9	75.00%
Total	16	31	23	74.20% ± 3.91%

**Table 2:** Details of the aggregates number count are reported for untreated mutant p.R239C embryos.. Means and standard deviations are described at the bottom the table.

Aggregates Mean Number of Untreated Mutant p.R239C Embryos			
Experiment	t0	t24	t48
n°1	55.60	59.60	64.00
n°2	58.00	63.67	67.67
n°3	53.60	59.40	61.60
Mean ± SD	55.88 ± 11.43	61.06 ± 15.36	64.63 ± 19.94

**Table 3:** Details of the aggregates number count are reported for treated mutant p.R239C embryos. Means and standard deviations are described at the bottom the table.

Aggregates Mean Number of Treated Mutant p.R239C Embryos			
Experiment	t0	t24	t48
n°1	55.44	51.33	19.33
n°2	58.45	52.09	25.91
n°3	52.80	48.40	21.80
Mean ± SD	55.57 ± 11.17	50.61 ± 10.73	22.35 ± 19.27

**Table 4:** For each experiment, among the total number of shocked embryos (“shocked”), embryos presenting a decreased number of aggregates (“rescued”) and the ones not showing any beneficial effects (“not rescued”) are shown in table. The rescued are represented in terms of percentage (“% rescue”) with respect to the total number of shocked embryos. Percentages of mean value and standard deviation are shown at the bottom of the table.

Number of Shocked Mutant p.R239C Embryos				
Experiment	Untreated	Shocked	Rescued	% Rescued
n°1	5	6	4	66.6%
n°2	4	5	4	80.0%
n°3	5	7	6	85.7%
Total	14	18	14	77.40% ± 9.81%

**Table 5:** Aggregate count number details in unshocked mutant p.R239C embryos, with the mean and the respective SD reported at t0, t3, and t24.

Aggregates Mean Number of Unshocked Mutant p.R239C Embryos			
Experiment	t0	t3	t24
n°1	53.20	54.80	58.80
n°2	57.00	63.25	67.00
n°3	56.80	64.20	68.80
Mean ± SD	55.57 ± 8.36	60.57 ± 11.24	64.71 ± 15.19

**Table 6:** Aggregate count number details in shocked mutant p.R239C embryos, with the mean and the respective SD reported at t0, t3, and t24.

Aggregates Mean Number of Shocked Mutant p.R239C Embryos			
Experiment	t0	t3	t24
n°1	47.67	35.50	39.67
n°2	50.80	30.00	35.20
n°3	51.43	26.43	32.71
Mean ± SD	49.97 ± 9.95	30.64 ± 11.65	35.86 ± 11.27

## Supplementary Materials

The following are available online at https://www.mdpi.com/2073-4425/11/12/1490/s1: Figure S1. Aggregates quantification; Figure S2. Tests performed to evaluate ethanol and CEF healthy concentration on embryos; Figure S3. Effect of ceftriaxone (CEF) 1.0 mM on gfap promoter; Figure S4. Effects of thermal shock on p.R239C aggregates; Figure S5. Electron microscope analysis on wildtype embryo; Table S1. Effects of ceftriaxone (CEF) on p.R239C mutant embryos in one of the experiments, here shown as exemplary; Table S2. Effect of ceftriaxone (CEF) 1.0 mM on GFAP WT-GFP embryos.
