# Supplementary material for: Alexander Disease Modeling in Zebrafish: An In Vivo System Suitable to Perform Drug Screening
Source: Genes (Basel). 2020 Dec 11;11(12):1490. doi: 10.3390/genes11121490 (PMC7764705; doi:10.3390/genes11121490)
Supplement: Supplementary file 1 [file genes-11-01490-s001.pdf]

Supplementary materials

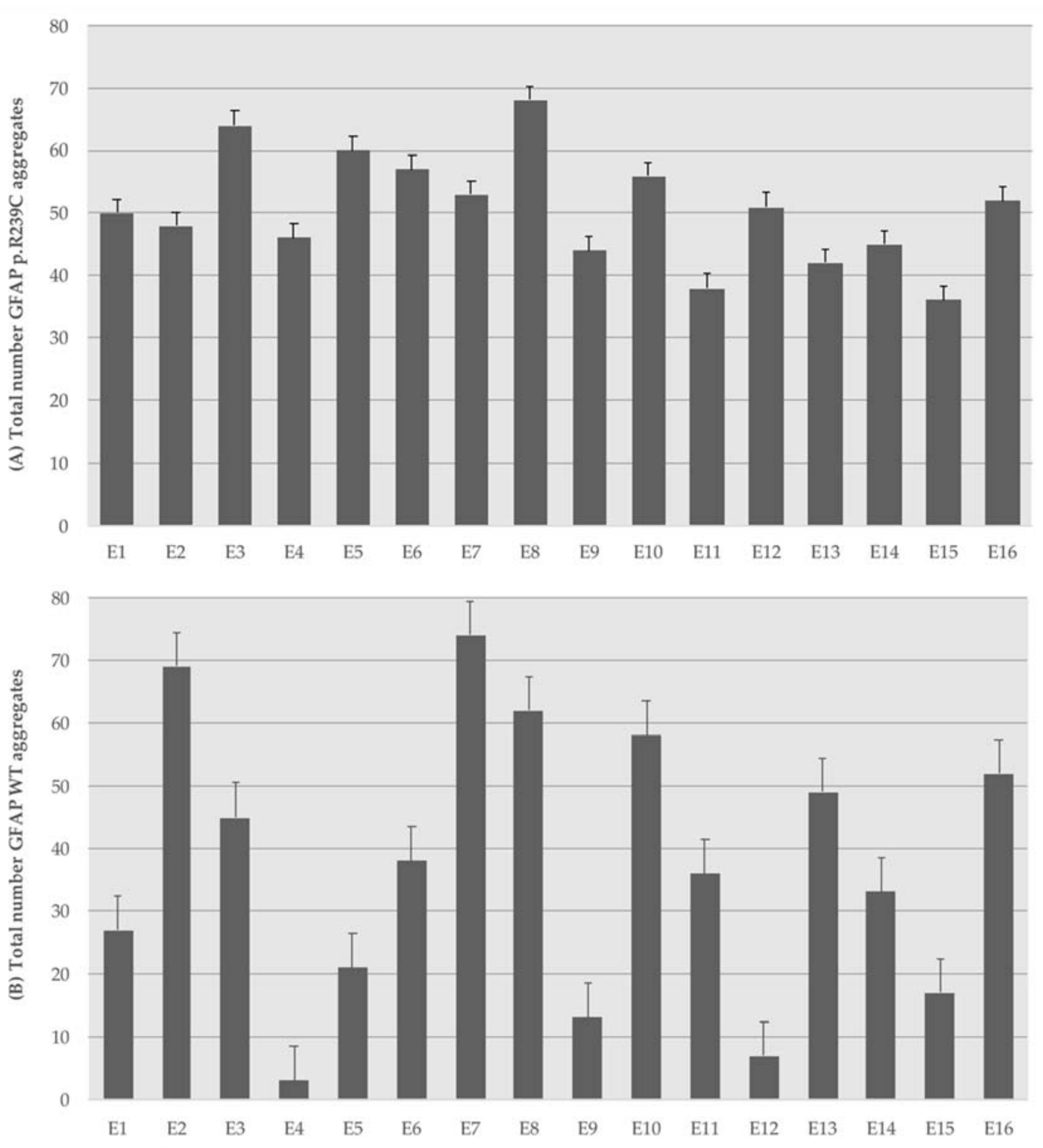

Figure S1: Aggregates quantification. The histograms represent the number of aggregates detected in **(A)** 16 embryos GFAP R239C-GFP and in **(B)** 16 embryos GFAP WT-GFP at 24 hpf, respectively. The count of the aggregates has been made by ImageJ software 1.52q and processed with Microsoft Office® Excel.

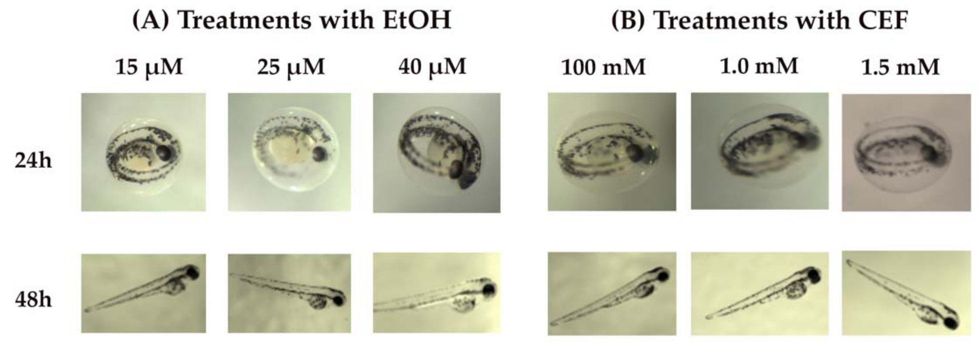

Figure S2. Tests performed to evaluate ethanol and CEF healthy concentration on embryos. Embryos have been treated for 48 hours with different concentrations of **(A)** ethanol at 15 μM , 25 μM and 40 μM . **(B)** Embryos treated with CEF at 100 mM, 1.0 mM and 1.5 mM.

**(A) GFAP p.R239C Untreated**	
**Embryo**	**t48**
E41	↔
E42	↔
E44	↔
E45	↔
E46	↔
E47	↔
E48	NO
E50	↓
E53	↔
E55	↔

**(B) GFAP p.R239C + CEF 1.0 mM**	
**Embryo**	**t48**
E57	↓
E58	NO
E59	NO
E61	↔
E65	↔
E66	NO
E67	↓
E68	NO
E71	↔
E72	↓

Table S1. Effects of ceftriaxone (CEF) on p.R239C mutant embryos in one of the experiments, here shown as exemplary. **(A)** The table represents the presence of aggregates in 10 untreated mutated embryos at t0 and after 48 hours (t48). **(B)** Effect of CEF 1.0 mM, at the same exposition time, on aggregates of 10 mutated p.R239C embryos. (↔: amount of aggregates not changed, ↓: aggregates decreased, NO: no aggregates disappeared, E: embryo, followed by the code number).

(A) GFAP WT-GFP Untreated	
**Embryo**	**t48**
E2	↔
E5	↓
E7	↓
E9	↔
E13	↔
E15	↔

(B) GFAP WT-GFP + CEF 1.0 mM	
**Embryo**	**t48**
E17	↔
E24	↓
E29	↔
E30	↔
E36	↓

Table S2. Effect of ceftriaxone (CEF) 1.0 mM on GFAP WT-GFP embryos. In table **(A)** is represented the variation of aggregates number in 6 untreated GFAP WT-GFP embryos at 24 hpf and after 48h (t48), while in table **(B)** is shown the variation of aggregates number in 5 GFAP WT-GFP embryos after 48 hours of treatment with CEF 1.0 mM, from 24 hpf to 72 hpf (t48) (↔: amount of aggregates not changed, ↓: aggregates decreased, NO: no aggregates disappeared, E: embryo, followed by the code number).

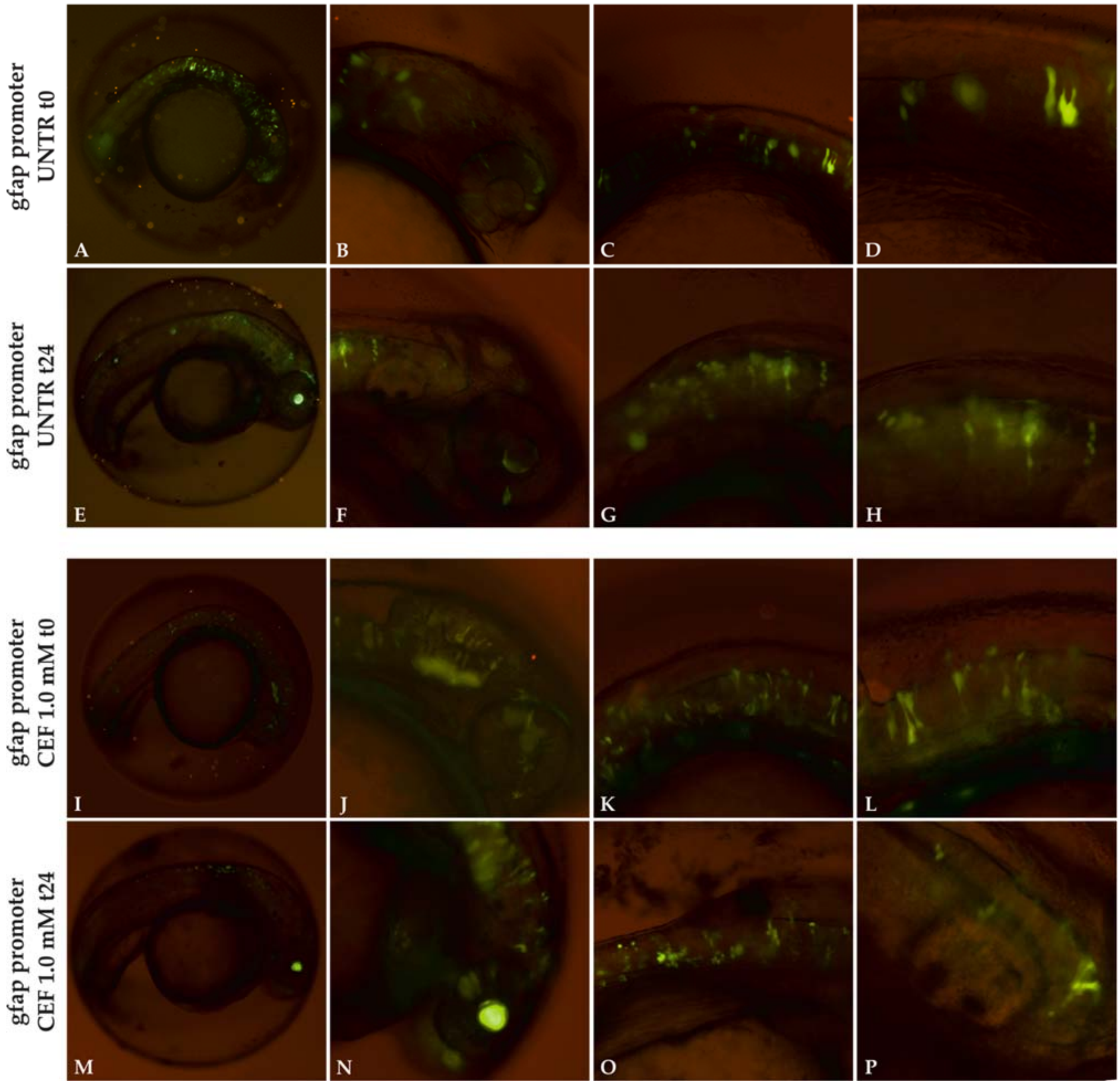

Figure S3. Effect of ceftriaxone (CEF) 1.0 mM on *gfap* promoter. Untreated embryos expressing *gfap* promoter as filamentous structures from **(A)** to **(H)** at 24 and 48 hpf, respectively. From **(M)** to **(P)** the effect of CEF 1.0 mM treatments on *gfap* promoter in embryos at 48 hpf, respect to t0 from **(I)** to **(L)** at 24 hpf. Magnification: 4X (A, E, J, M); 10X (B, C, F, G, J, K, N, O) and 20X (D, H, L, P).

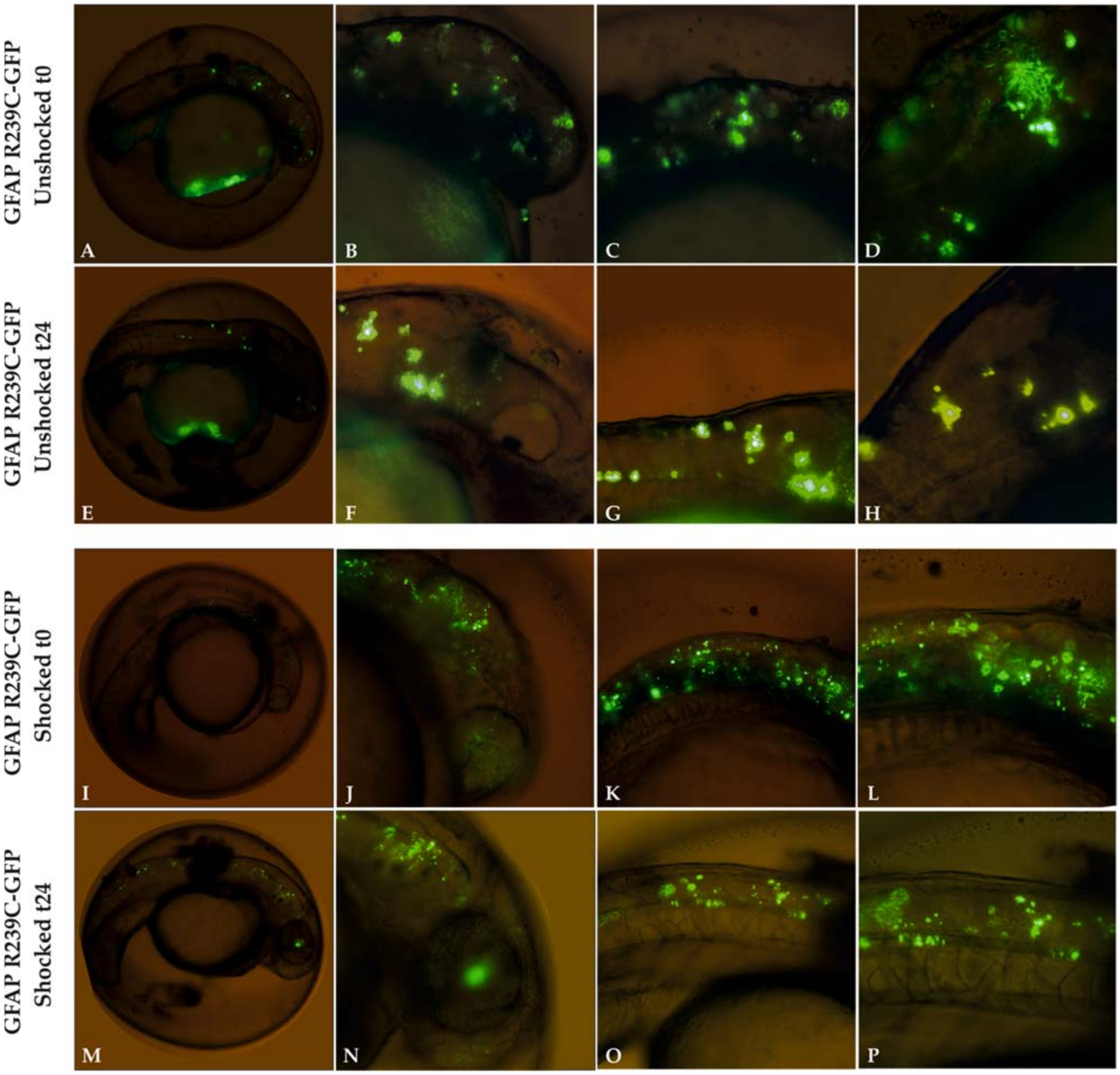

Figure S4. Effects of thermal shock on p.R239C aggregates. In figures from **(A)** to **(H),** mutated embryos showing diffuse aggregates formation at 24 hours after microinjection (t0) and after 24 hours. Figures from **(I)** to **(L)** show GFAP p.R239C aggregates in embryos at 24 hours after microinjection (t0), while figures **(M)** to **(P)** show the reduction of GFAP p.R239C aggregates in the same embryo at 48 hours after the end of the thermal sock (t48). Magnification: 4X (A, E, J, M); 10X (B, C, F, G, J, K, N, O) and 20X (D, H, L, P).

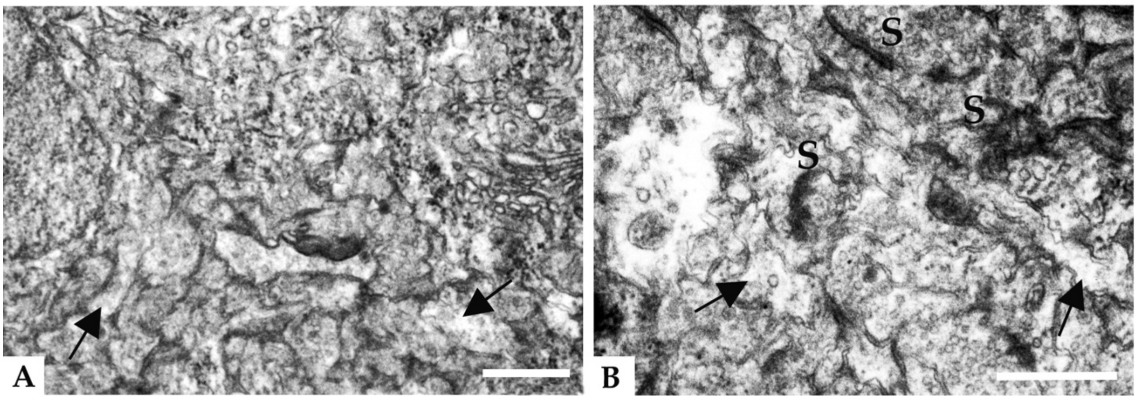

Figure S5. Electron microscope analysis on wildtype embryo. In **(A)** and **(B)** an electron micrograph of the neuropil region in the telencephalon of a 5 dpf wildtype embryo, not-injected with GFAP WT plasmid. No such electron-dense material in glial processes, identified by their irregular shape (arrows), is detected in the neuropil. Scale bar: 500 nm. (S: pre-synaptic bulbs).
